# Chronic Resveratrol Treatment Protects Pancreatic Islets against Oxidative Stress in db/db Mice

**DOI:** 10.1371/journal.pone.0050412

**Published:** 2012-11-30

**Authors:** Young-Eun Lee, Ji-Won Kim, Eun-Mi Lee, Yu-Bae Ahn, Ki-Ho Song, Kun-Ho Yoon, Hyung-Wook Kim, Cheol-Whee Park, Guolian Li, Zhenqi Liu, Seung-Hyun Ko

**Affiliations:** 1 Division of Endocrinology and Metabolism, Department of Internal Medicine, The Catholic University of Korea, Seoul, Korea; 2 Division of Nephrology, Department of Internal Medicine, The Catholic University of Korea, Seoul, Korea; 3 Division of Endocrinology and Metabolism, Department of Medicine, University of Virginia Health System, Charlottesville, Virginia, United States of America; University of Bremen, Germany

## Abstract

Resveratrol (RSV) has anti-inflammatory and anti-oxidant actions which may contribute to its cardiovascular protective effects. We examined whether RSV has any beneficial effects on pancreatic islets in db/db mice, an animal model of type 2 diabetes. The db/db and db/dm mice (non-diabetic control) were treated with (db-RSV) or without RSV (db-control) (20 mg/kg daily) for 12 weeks. After performing an intraperitoneal glucose tolerance test and insulin tolerance test, mice were sacrificed, the pancreas was weighed, pancreatic β-cell mass was quantified by point count method, and the amount of islet fibrosis was determined. 8-Hydroxydeoxyguanosine (8-OHdG), an oxidative stress marker, was determined in 24 h urine and pancreatic islets. RSV treatment significantly improved glucose tolerance at 2 hrs in db/db mice (P = 0.036), but not in db/dm mice (P = 0.623). This was associated with a significant increase in both pancreas weight (P = 0.011) and β-cell mass (P = 0.016). Islet fibrosis was much less in RSV-treated mice (P = 0.048). RSV treatment also decreased urinary 8-OHdG levels (P = 0.03) and the percentage of islet nuclei that were positive for 8-OHdG immunostaining (P = 0.019). We conclude that RSV treatment improves glucose tolerance, attenuates β-cell loss, and reduces oxidative stress in type 2 diabetes. These findings suggest that RSV may have a therapeutic implication in the prevention and management of diabetes.

## Introduction

Diabetes mellitus has become a major health issue worldwide due to its high prevalence and its associated morbidity and mortality. Though insulin resistance has been identified as the early event in the pathogenesis of type 2 diabetes, pancreatic β-cell dysfunction plays a pivotal role in the disease development [1], [2]. Strong evidence has demonstrated that β-cell dysfunction is closely related to oxidative stress due to increased reactive oxygen species (ROS) generation [3], [4], [5], [6], [7] and possibly also weakened antioxidant defense in pancreatic islets during chronic hyperglycemia. This oxidative imbalance results in decreased insulin secretory capacity and β-cell viability, each contributing to β-cell failure and the onset of diabetes [3], [4]. Several mechanisms, including increased polyol pathway flux, increased advanced glycation end products (AGE) formation, activation of protein kinase C and mitochondrial dysfunction, have been shown to contribute to hyperglycemia-induced ROS generation [5], with subsequent activation of various stress pathways, such as NF-kB, JNK/SAPK, and p38 MAPK. On the other hand, hyperglycemia reduces anti-oxidant enzymes and inhibits their enzymatic activities, which further increases the overall oxidative environment prevalent in diabetes [6], [7].

Resveratrol (3,4′,5-trihydroxystilbene, RSV) is a naturally occurring polyphenolic phytoalexin produced by certain spermatophytes in response to injury [8], [9], [10]. The importance of this non-flavonoid class of polyphenolic compound was unraveled when several epidemiologic studies showed a significant inverse relationship between wine consumption and the incidence of cardiovascular risk, also called the “French Paradox” [11], [12]. It was suggested that polyphenols present in plants are one of the main components responsible for the cardiovascular protection conveyed by fruits and vegetables, and RSV is considered as the key candidate component. RSV is found in the skin of red grapes, wine, apples, peanuts, blueberries and cranberries, and it has been shown to have anti-apoptosis, anti-inflammatory, anti-aging and anti-cancer effects as well as cardiovascular protective effects both *in vivo* and *in vitro* [13],[14]. Recent studies also showed that RSV *in vitro* attenuates cellular oxidative stress, and protects endothelial cells from oxidative stress-induced apoptosis [15], [16].

Previous studies have demonstrated that RSV has anti-diabetic effects. It decreases blood glucose levels in animal models of type 1 or type 2 diabetes mellitus [17], [18], reduces insulin secretion in animals with hyperinsulinemia, inhibits cytokine actions and attenuates oxidative damage in pancreatic tissue [19]. Thus, RSV may exert beneficial actions on pancreatic β-cells in diabetes, likely through its anti-oxidant actions. However, studies on the antioxidant effects of resveratrol are very limited in animal models of type 2 diabetes and there has been no report on the effects of chronic resveratrol treatment on the morphologic changes of pancreatic islets.

In the current study, we aimed to investigate whether chronic administration of RSV protects pancreatic islets and improves glycemia in db/db mice, a well-accepted and most widely used mouse model of type 2 diabetes. The *db* gene encodes a G-to-T point mutation of the leptin receptor, which causes a defect affecting hypothalamic responses and leads to the development of hyperphagia, obesity, hyperlipidemia, hyperinsulinemia, insulin resistance, and diabetes [20], [21]. Our results suggest that chronic treatment of diabetic animals with resveratrol improves glucose tolerance, attenuates β-cell loss and reduces oxidative stress.

## Materials and Methods

### Animal experiments

Male, 5-week-old db/db and db/dm (non-diabetic control) mice were obtained from Charles River Japan (Kanagawa, Japan). The animals received an intraperitoneal glucose tolerance test and insulin tolerance test (baseline, see below) and were then treated randomly with either resveratrol (RSV, 20 mg/kg/day, Sigma, St. Louis, MO) or vehicle (0.5% methylcellulose, Sigma) orally by gavage tube for 12 weeks. A total of four groups of mice were included: (1) db/dm mice with vehicle (dm-control) (n = 8), (2) db/dm mice with resveratrol (dm-RSV) (n = 8), (3) db/db mice with vehicle (db-control) (n = 8), and (4) db/db mice with resveratrol (db-RSV) (n = 8). Mice were maintained under standard housing conditions at 22±2°C with a 12-h light/dark cycle, a standard chow fed *ad libitum* (Pico 5053, LabDiet, Brentwood, MO), and free access to tap water. Resveratrol at the dose selected has been shown to attenuate oxidative stress in db/db mice [22].

Body weight, the amount of food and water consumption, and the blood glucose concentrations were monitored weekly. The dosage of resveratrol was adjusted weekly based on the body weight change. At the end of the 12-week intervention, the mice were sacrificed after receiving additional intraperitoneal glucose and insulin tolerance tests (one day apart after an 8-h fast). The pancreas of each mouse was harvested, weighted, and fixed with 4% formaldehyde for histological examination.

### Ethics Statement

The study was carried out in strict accordance with the recommendations of the Guide for the Care and Use of Laboratory Animals of the National Institutes of Health. The protocol was approved by the Laboratory Animal Care Committee at the Catholic University of Korea (Permit Number; CUMC-2010-0064).

### Glucose and Insulin Tolerance Tests

After 8 h of fasting, glucose tolerance test was performed. Glucose solution (2 g/kg body weight) was administered intraperitoneally, and the blood glucose was measured from tail snipping at 0, 30, 60, 90, and 120 min after the initial glucose loading. Blood glucose was determined using a glucometer (Roche, Mannheim, Germany). The area under the curve of the glucose concentrations (AUCg) was calculated. For the insulin tolerance test, mice were also fasted for 8 h and then given a human insulin (Novolin R) injection intraperitoneally at a dose of 0.4 unit/g body weight [23]. Blood glucose measurements were obtained from tail snipping at 0, 3, 6, 9, 12, 15, 18, 21, 24, 27, and 30 min, and the insulin sensitivity index Kitt (%/min) was calculated. Plasma insulin levels were measured using an insulin radioimmunoassay kit (Millipore, USA).

### Immunohistochemical Staining

The pancreatic tissue samples were fixed in 4% formaldehyde, dehydrated in a graded series of ethanol concentrations, and embedded. For immunochemistry analysis, paraffin-fixed tissue sections were incubated overnight at 4°C with anti-insulin antibody (1∶500, Linco Research Inc., St. Charles, MO), washed, and then incubated with anti-HRP (1∶200, Zymed Laboratories Inc., San Francisco, CA) at room temperature for 30 min. The sections were then developed with DAB, counterstained with hematoxylin, and examined using an Olympus AX70 microscope (Olympus, Japan).

For the immunostaining of oxidative stress-related proteins, a monoclonal antibody to 8-hydroxy-2′-deoxyguanosine (8-OHdG, 1∶100, Abcam, Cambridge, MA), a marker of oxidative stress-induced DNA damage, was used. The sections were treated with microwave irradiation for 15 min in 10 mM citric buffer (pH 6.0) for antigen retrieval. After incubation with horse serum for 30 min to block non-specific reactions, the primary antibody was applied overnight at 4°C. They were then incubated with biotinylated horse anti-mouse IgG antibody with peroxidase-conjugated streptavidin labeling reagent (1∶100; Vector Laboratories, Burlington, ON, Canada) as the secondary antibody. The number of 8-OHdG-positive nuclei and total nuclei (positive and negative) of islets were counted in each pancreatic section. On average, 19.4±11.8 non-overlapping islets per pancreas were systematically measured.

### Masson’s Trichrome Staining

To examine the amount of islet fibrosis, sections were stained using Masson’s trichrome method. To calculate the percentage of blue-stained (fibrosis) areas within islets, each section was processed by planimetry using an image analyzer (Optimas 6.51, Media Cybertics, Tempe, AR). The amount of fibrosis was calculated from a mean of 24.1 non-overlapping islets per pancreas and was presented as a percentage of the total islet area: (area of fibrosis/total area of islets) X 100.

### β-Cell Mass in Pancreatic Islet

The relative β-cell volumes were measured by the point counting method [24] using an Olympus AX70 microscope connected to a camera equipped with a color monitor with 100-point transparent overlay. Briefly, pancreas sections stained with anti-insulin antibody were visualized under 200 X magnification and positioned under a regular lattice overlaid on a color monitor. The relative β-cell volume in the pancreatic tissue was described as the number of points corresponding to the anti-insulin antibody-stained area/number of points corresponding to remaining pancreatic area. β-Cell mass was calculated by multiplying the relative percentage of β-cells by the total pancreatic weight [25]. An average of 207.8 fields and 20784.4 points in non-overlapping fields were counted systematically from each section with 5 sections being selected per tissue block.

### Plasma and Urinary ROS Markers

Urine samples were collected using metabolic cages. After centrifugation at 13,000 rpm for 30 min, we measured isoprostane (Urinary 8-Epiprostaglandin-F2a assay EIA kit, OXIS Health Products, Inc., Portland, OR) and 8-OHdG (8-Hydroxy-2-deoxy Guanosine EIA kit, OXIS Health Products), two oxidative stress markers [26], according to the manufacturer’s instructions. For measurement of 8-OHdG, samples and standards were added into a 96-well plate, incubated with primary antibody at 37°C for 1 h and then incubated with secondary antibody at 37°C for 1 h. Chromogen and the stop solution were then added sequentially. For measurement of isoprostane, sample mixtures and standards were added into a 96-well plate. After incubation with the respective primary and secondary antibodies, TMB substrate was added. After the stop solution was added, the absorbance was read at 450 nm and the concentration was determined.

### Data Analysis

Data are presented as the mean ± SE and were analyzed using SPSS statistical software (SPSS 11.0). Comparisons were performed by one-way analysis of variance or unpaired Student’s *t* test as appropriate. *P*<0.05 was considered statistically significant.

## Results

### Effect of Resveratrol on the Glucose Tolerance Test and Insulin Sensitivity

The body weight of db/db mice was significantly higher than that of the lean (db/dm) littermates. Chronic administration of RSV did not produce any significant changes in body weight compared to those placebo-treated control mice, both in db/db (db-RSV vs. db-control, 41.3±1.4 g vs. 40.5±1.7 g, P = 0.724) and db/dm mice (dm-RSV vs. dm-control, 30.3±0.7 g vs. 30.2±0.6 g, P = 0.924) at 12 weeks.

**Figure 1:**
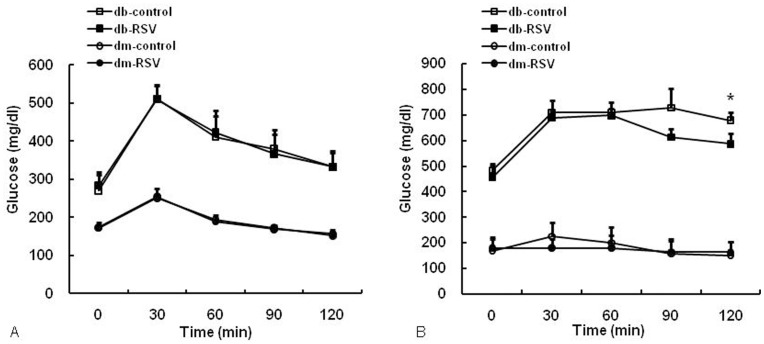
Results of an intraperitoneal glucose tolerance test. Mice received vehicle (control) or resveratrol (20 mg/kg daily) orally for 12 weeks. The glucose concentration was determined at the time point indicated after glucose challenge (2 g/kg body weight) at baseline (A) and 12 weeks (B). Data are the mean ± SE. n = 8 in each group. *P<0.05 vs. vehicle-treated control group.

**Figure 2:**
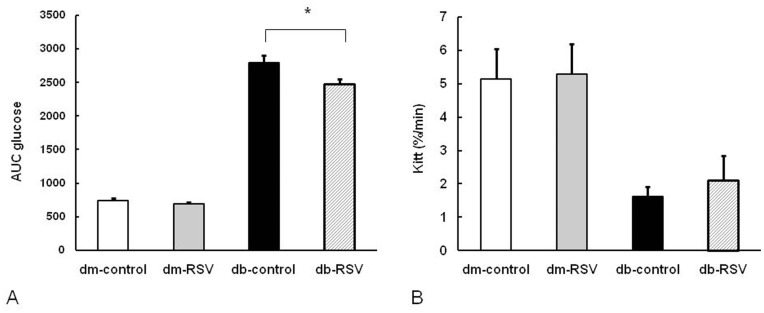
Effect of oral administration of resveratrol on the area under the curve of glucose (AUCg) during a glucose tolerance test and insulin tolerance test. (A) AUCg during 2h glucose tolerance test. (B) K*itt* values (%/min) from the insulin tolerance test at 12 weeks as a surrogate insulin sensitivity index. Data are the mean ± SE. n = 8 in each group. *P<0.05 vs. vehicle-treated control group.

The effects of RSV on glucose tolerance in both the control and experimental groups are shown in Fig. 1. Db/db mice had significantly higher fasting blood glucose than db/dm mice at baseline and there were no differences between the controls and the RSV-treated mice (283.5±24.8 mg/dL vs. 270.3±29.1 mg/dL, db-RSV vs. db-control, P = 0.734, Fig. 1A). Treatment of db/db mice with RSV for 12 weeks significantly improved the glucose tolerance of db/db mice, resulting in a significantly lower blood glucose concentrations at 120 min after glucose loading (560.3±26.6 mg/dL vs. 688.0±23.8 mg/dL, db-RSV vs. db-control, P = 0.004, Fig. 1B). However, there were no significant differences in blood glucose levels at 120 min after glucose loading between RSV-treated db/dm mice and control db/dm mice (163.3±14.1 mg/dL vs. 151.4±18.9 mg/dL, dm-RSV vs. dm-control, P = 0.623) or in the fasting glucose concentrations between RSV-treated db/db mice and control db/db mice (456.6±12.4 mg/dL vs. 484.0±17.4 mg/dL, db-RSV vs. db-control, P = 0.460, Fig. 1B) at 12 weeks. The mean value of the AUCg was also significantly lower in the db-RSV group than in the db-control group during the glucose tolerance test (2476.4±74.4 mg/dL vs. 2797.3±106.7 mg/dL, db-RSV vs. db-control, P = 0.036). Again, no statistically significant difference in the AUCg was detected between the dm-control and the dm-RSV group at 12 weeks (695.1±19.6 mg/dL vs. 743.3±27.3 mg/dL, dm-RSV vs. dm-control, P = 0.620, Fig. 2A).

We obtained K*itt* values (%/min) from the insulin tolerance test at 12 weeks as a surrogate insulin sensitivity index. K*itt* levels were not significantly different between RSV and the control groups in either db/dm mice (5.29±0.9%/min vs. 5.14±0.9%/min, dm-RSV vs. dm-control, P = 0.893) or db/db mice (2.11±0.75%/min vs. 1.63±0.3%/min, db-RSV vs. db-control, P = 0.564, Fig. 2B).

The plasma insulin concentrations were not different between the RSV-treated and control db/db mice (0.34±0.01 ng/mL vs. 0.36±0.01 ng/mL, db-RSV vs. db-control, P = 0.591).

### Effect of Resveratrol on β-cell Mass and Islet Fibrosis

**Figure 3:**
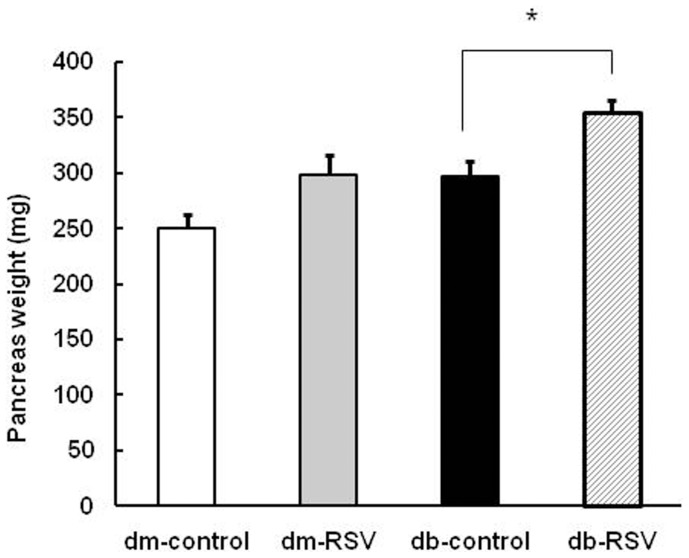
Effects of long-term resveratrol treatment on pancreas weight. Data are the mean ± SE. n = 8 in each group. *P<0.05 vs. vehicle-treated control group.

**Figure 4:**
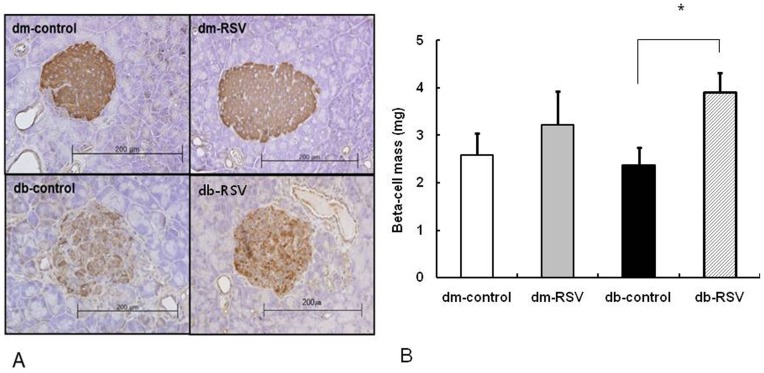
Effect of long-term administration of resveratrol on pancreatic β-cell mass. (A) Immunostaining of pancreatic islets using an insulin antibody (x 200). (B) Quantification of β-cell mass. *P<0.05 vs. vehicle-treated db-control group. n = 8 in each group.

Resveratrol treatment for 12 weeks significantly increased the pancreas weight in db/db mice (355.3±12.0 mg vs. 296.7±14.6 mg, db-RSV vs. db-control, P = 0.011, Fig. 3). Though the pancreas weight tended to be higher in RSV-treated db/dm mice than in db/dm control mice, this increase was not statistically significant (P = 0.055). Compared to the nondiabetic control group (dm-control and dm-RSV), the pancreatic islet architecture of the db/db mice (db-control) was disorganized and the insulin-stained area was markedly decreased. In RSV-treated db/db mice, however, islet destruction and the insulin-stained area were more preserved than those of the db/db mice (Fig. 4A). Similarly, RSV significantly increased β-cell mass in db/db (3.9±0.4 mg vs. 2.4±0.4 mg, db-RSV vs. db-control, P = 0.016, Fig. 4B) but not in db/dm mice (3.2±0.7 mg vs. 2.6±0.5 mg, dm-RSV vs. dm-control, P = 0.461).

**Figure 5:**
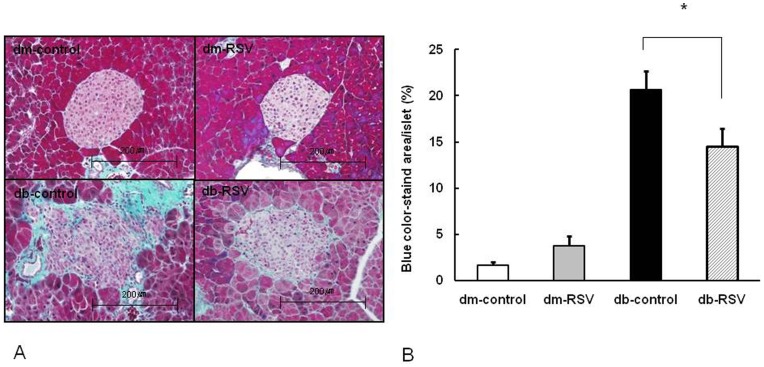
Effect of resveratrol on islet fibrosis. (A) Representative Masson’s trichrome staining from pancreatic tissue (x 200). (B) Quantification of islet fibrosis. Data are the mean ± SE. *P<0.05 vs. vehicle-treated db-control group. n = 8 in each group.

The degree of islet fibrosis was measured using trichrome staining. The RSV treatment did not affect islet morphology or fibrosis in db/dm mice. As expected, the islets were enlarged and architecturally disorganized in db/db mice compared to db/dm mice at 12 weeks (Fig. 5A). RSV treatment significantly attenuated the blue-colored fibrosis area in islets and partially restored the islet size and architecture in db/db mice (Fig. 5A). Accordingly, the islet fibrosis area was markedly reduced with RSV treatment in db/db mice at 12 weeks (14.5±2.0% vs. 20.6±2.1%, db-RSV vs. db-control, P = 0.048, Fig. 5B).

**Table 1:** Measurement of urinary ROS marker generation.

	dm-control	dm-RSV	db-control	db-RSV
Urinary isoprotane (ng/day)	4.9±1.3	4.6±1.0	12.8±2.3*	6.0±1.3**
Urinary 8-OHdG (ng/day)	13.0±4.2	14.9±9.2	84.6±4.4*	37.3±4.9**

Values are given as the mean ± SE for groups (n = 8 for each). *P<0.005 vs. dm-control; **P<0.05 vs. db-control group.

**Figure 6:**
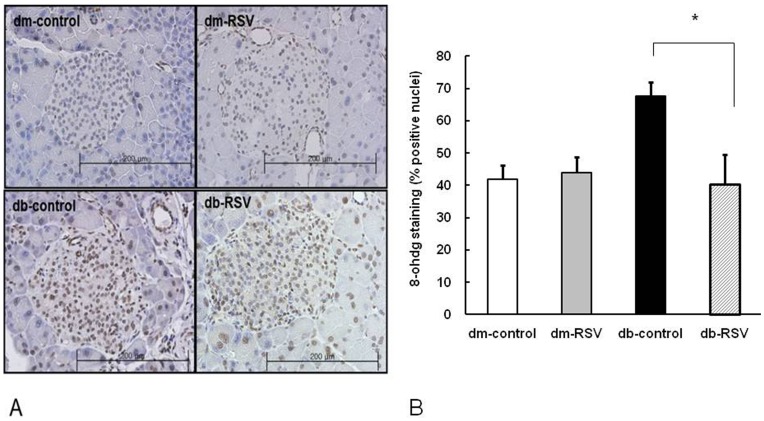
Immunostaining of pancreatic tissue. (A) 8-OHdG staining (x 200). (B) Measurement of 8-OHdG positive nuclei (%) in the pancreatic islets of db/db mice. Mice received either vehicle (control) or resveratrol (20 mg/kg/day) for 12 weeks. Data are the mean ± SE. n = 8 in each group. *P<0.05 vs. vehicle-treated control group.

### Effect of Chronic RSV Treatment on ROS Generation

As shown in Table 1, db/db mice showed markedly higher urinary isoprostane and 8-OHdG levels than those of control db/dm mice (P<0.005 for both). RSV treatment for 12 weeks reduced urinary isoprostane back to the control levels (P<0.05) and urinary 8-OHdG levels by >50% (P<0.05).

Similar to urinary ROS markers, 8-OHdG immunostaining of pancreatic sections demonstrated that islet expression of 8-OHdG was significantly higher in diabetic db/db mice than that of control non-diabetic db/dm mice (41.9±4.2 ng/day vs. 67.6±4.4 ng/day, dm-control vs. db-control, P<0.005, Fig. 6). RSV treatment for 12 weeks effectively abolished the increased expression of islet 8-OHdG and brought the percentage of 8-OHdG-positive nuclei in the pancreatic islets of db/db mice back to the levels observed in the control mice (40.5±9.2% vs. 67.6±4.4%, db-RSV vs. db-control, P = 0.019, Fig. 6B). RSV treatment did not impact the islet expression of 8-OHdG in nondiabetic control mice.

## Discussion

The current study demonstrates that chronic treatment with RSV significantly improves glucose tolerance, reduces oxidative stress, attenuates pancreatic islet fibrosis and preserves islet mass in an animal model of type 2 diabetes. These findings are important because pancreatic β-cell dysfunction, characterized by decreased insulin secretory capacity due to insufficient β-cell mass and/or functional defects of the β-cells [27], plays a pivotal role in the pathogenesis of type 2 diabetes [28].

Resveratrol has been extensively studied for its cardiovascular benefits, which are mediated by its polyphenols [11], [12]. After oral ingestion, resveratrol is rapidly absorbed into the blood circulation and distributed to various tissues and organs, including the pancreas, most likely in the forms of glucuronide and sulfate conjugate [14]. The possible mechanisms by which RSV exerts its cardiovascular protection involve inhibition of platelet aggregation, arterial vasodilation, favorable changes in lipid metabolism, antioxidant effects, stimulation of angiogenesis, induction of cardioprotective proteins, and insulin sensitization [13], [29].

Recent studies in rodents have demonstrated that RSV also possesses anti-hyperglycemic properties. RSV administration (0.5 mg/kg/day) for 2 weeks results in a dose-dependent lowering of the plasma glucose and lipid concentrations in streptozotocin and nicotinamide-induced diabetic rats [30]. Longer use of RSV (10 mg/kg/day for 8 weeks) reduces dyslipidemia, insulin resistance, hyperleptinemia and hypertension in obese Zucker rats [17]. RSV has also been shown to reverse hyperglycemia and improve insulin sensitivity in high-fat-induced obese rodents and in a type 1 diabetic animal model [31], [32]. Though the underlying mechanisms remain unclear, evidence suggests that RSV may exert these actions via the inhibition of the activities of IκB kinase-β (IKKβ), NF-κB, and protein kinase C [33], and/or the activation of Sirt1 and AMPK [19], [22], [34].

Our results clearly indicate that chronic administration of RSV has a protective effect on pancreatic islets, possibly via decreased oxidative stress both at the systemic level and also within the islets in type 2 diabetes. Indeed, both the 2 h blood glucose and AUCg levels during GTT were significantly decreased in RSV-treated diabetic mice, which were associated with increased β-cell mass, preserved islet architecture and decreased islet fibrosis. Thus, our evidence strongly suggests that RSV has a glucose-lowering effect and protects β-cells against metabolic stress. These beneficial effects may result from reduced oxidative stress associated with RSV treatment. Ample studies have convincingly demonstrated that RSV has potent antioxidant effects, including the scavenging of ROS and up-regulating the antioxidant enzyme expression in vascular endothelial cells [16], [22], [35], [36]. Our findings are also in line with *in vitro* studies using pancreatic β-cell lines (RIN-5F cells) in that resveratrol protected the cells from advanced glycation end product-induced oxidative stress and apoptosis [37]. Though we did not examine the mechanisms underlying resveratrol-induced reduction of ROS production, many other studies have demonstrated that RSV exerts beneficial effects on the sources of superoxide anion production, including NADPH oxidase, xanthine oxidase, the mitochondrial respiratory chain, and the arachidonic acid cascade (including lipoxygenase and cyclooxygenase) [38]. Resveratrol is also able to induce cellular antioxidants and phase 2 enzymes, thus reducing hydrogen peroxide generation, levels of oxidized glutathione reductase and MPO activities [39]. Indeed, chronic resveratrol treatment of db/db mice for 5 weeks (0.04% in diet) suppressed blood glucose elevation, improved dyslipidemia, and lowered the serum lipid peroxide (TBARS) concentration [37]. At a higher dose (0.3% with chow for 8 weeks), resveratrol attenuated renal injury and enhanced mitochondrial biogenesis in the kidney of db/db mice through the normalization of Mn-SOD function and glucose-lipid metabolism [40].

In the current study, we found that RSV treatment decreased oxidative markers in both urine and pancreatic islets in type 2 diabetic mice. In response to hyperglycemia, islets exhibit increased oxidative stress [3], [41], likely resulting from an increased production of mitochondrial ROS, nonenzymatic glycation of proteins, and glucose autoxidation [5], [6], [7]. Elevated oxidants and markers for oxidative tissue damage, such as hydroperoxides, oxidation of DNA bases, 8-epi-prostaglandin F2a, and 8-hydroxydeoxyguanine, have been reported in patients with diabetes [42]. Oxidative stress causes tissue damage in the pancreas, and the extent of damage correlates with the loss of β-cell mass [43]. Given the pivotal role of oxidative stress in islet destruction and dysfunction in diabetes, it is entirely possible that RSV can protect pancreatic islets by attenuating hyperglycemia-induced oxidative stress within islets. Although resveratrol may have exerted other salutary effects on the islets in addition to its antioxidative action, our results suggest that its antioxidative effects are at least partially responsible for the observed improvement in islet mass and function in resveratrol-treated db/db mice. Indeed, other antioxidant treatments have shown to protect β-cells in insulin resistant rodents. In β-cell lines or isolated rat islets, incubation with antioxidant N-acetyl cysteine or overexpression of antioxidant enzyme glutathione peroxidase prevented the deleterious effects of hyperglycemia on insulin gene expression. Moreover, antioxidant treatment of insulin resistant rodents, such as Zucker diabetic fatty rats or OLETF rats, decreased markers of oxidative stress, improved insulin gene expression and glycemic control, and protected β-cells through the attenuation of both islet fibrosis and β-cell apoptosis [44], [45]. Moreover, db/db mice at 6–8 weeks old are usually hyperinsulinemic and hyperlipidemic. At 10–14 weeks old, db/db mice become fully diabetic with significant higher plasma glucose and oxidative stress [21]. Evidence suggests that in db/db mice, vascular superoxide production was not increased in 6 week-old mice, and plasma ROS levels seemed to increase after approximately 10 weeks of age [21], [46]. As such, we believe that RSV prevented rather than reversed pathophysiological changes in db/db mice. Taken together, our results strongly suggest that chronic oxidative stress plays a major role in hyperglycemia-induced β-cell failure and suggest the potential use of antioxidants such as resveratrol in diabetes prevention and/or therapy [47]–[50].

We did not examine the role of lipotoxicity in the current study. Because lipotoxicity also mediates damage to pancreatic islets and resveratrol reduces tissue lipid levels in other organs, resveratrol may have also reduced the levels of lipid in islets, and this may have contributed to the observed protection of β-cells in db/db mice. However, this possibility remains to be confirmed. In a separate study, we showed that incubation of pancreatic β-cells (INS-1 cells) with resveratrol prevented cellular accumulation of lipid when exposed to high concentrations of palmitate, oleate and glucose (data not shown).

In the current study, we did not observe any improvement in insulin sensitivity as calculated by Kitt values and plasma insulin concentrations with RSV treatment despite a significant increase in β-cell mass. This was not entirely surprising as the diabetic mice were significant hyperglycemic, which may have masked the insulin-sensitizing effect generated by RSV treatment. It remains likely that with more prolonged treatment or a higher dosage, especially on top of antihyperglycemic agent such as insulin to reduce glucose toxicity, insulin sensitivity may improve and fasting plasma insulin concentrations may decrease. Inasmuch as the plasma insulin concentrations and the Kitt values did not differ between the control and resveratrol-treated db/db mice, the significant improvement in glucose tolerance suggests an improvement in the β-cell response to glucose challenge in resveratrol-treated db/db mice. This is consistent with our observation that resveratrol decreased islet fibrosis and ROS and increased β-cell mass. As ROS plays important roles in the pathogenesis of insulin resistance, it is likely that longer treatment of db/db mice with reveratrol could improve insulin sensitivity as well. Indeed, treatment of high-fat diet obese mice for 16 weeks to 6 months all showed a reduced plasma insulin concentration and increased insulin sensitivity [32]. However, caution is needed, as resveratrol has been shown to potentiate glucose-stimulated insulin secretion in cultured INS-1 cells [51] and inhibit insulin secretion in rat pancreatic islets [52]. Direct evidences of resveratrol’s effect on pancreatic insulin content and insulin secretion in the db/db mouse model remain lacking. In addition, it is not surprising that changes in pancreas weight did not exactly match the changes in β-cell mass, as the islet weight accounts for only 1–2% of the pancreas weight and was calculated by multiplying the relative percentage of β-cells by the total pancreatic weight. The lower β-cell mass in db-control mice is consistent with increased β-cell loss.

In conclusion, chronic treatment with RSV improves glucose tolerance, attenuates high glucose-induced oxidative stress and preserves pancreatic β-cell mass in type 2 diabetic db/db mice. Together with its salutary cardiovascular benefits, resveratrol could be a promising therapeutic candidate for the prevention and treatment of diabetes and its associated cardiovascular complications. Further studies are needed to define the molecular and cellular mechanisms underlying resveratrol-mediated protective effects against oxidative stress and fibrosis in the islets.
